# Education Research: Competency-Based EEG Education: An Online Routine EEG Examination for Adult and Child Neurology Residents

**DOI:** 10.1212/NE9.0000000000200094

**Published:** 2023-10-16

**Authors:** Fábio A. Nascimento, Hong Gao, Roohi Katyal, Rebecca Matthews, Samantha V. Yap, Stefan Rampp, William O. Tatum, Roy E. Strowd, Sándor Beniczky

**Affiliations:** From the Division of Epilepsy (F.A.N.), Department of Neurology, Washington University School of Medicine, St. Louis, MO; Department of Neurology (F.A.N., S.V.Y.), Massachusetts General Hospital, Harvard Medical School, Boston; Department of Internal Medicine (H.G.), Wake Forest University School of Medicine, Winston-Salem, NC; Division of Epilepsy (R.K.), Department of Neurology, Louisiana State University Health Shreveport; Department of Neurology (R.M.), Emory University School of Medicine, Atlanta, GA; Department of Neurosurgery (S.R.), University Hospital Erlangen; Department of Neurosurgery (S.R.), University Hospital Halle (Saale), Germany; Department of Neurology (W.O.T.), Mayo Clinic, Jacksonville, FL; Department of Neurology (R.E.S.), Wake Forest University School of Medicine, Winston-Salem, NC; Department of Clinical Neurophysiology (S.B.), Danish Epilepsy Center, Dianalund and Aarhus University Hospital; and Department of Clinical Medicine (S.B.), Aarhus University, Denmark.

## Abstract

**Background and Objectives:**

We recently published expert consensus-based curricular objectives for routine EEG (rEEG) interpretation for adult and child neurology residents. In this study, we used this curriculum framework to develop and validate an online, competency-based, formative and summative rEEG examination for neurology residents.

**Methods:**

We developed an online rEEG examination consisting of a brief survey and 30 multiple-choice questions covering EEG learning objectives for neurology residents in 4 domains: normal, abnormal, normal variants, and artifacts. Each question contained a deidentified EEG image, displayed in 2 montages (bipolar and average), reviewed and optimized by the authors to address the learning objectives. Respondents reported their level of confidence (LOC, 5-point Likert scale) with identifying 4 categories of EEG findings independently: states of wakefulness/sleep, sleep structures, normal variants, and artifacts. Accuracy and item discrimination were calculated for each question and LOC for each category. The test was disseminated by the International League Against Epilepsy and shared on social media.

**Results:**

Of 2,080 responses, 922 were complete. Respondents comprised clinical neurophysiologists/experts (n = 41), EEG/epilepsy clinical fellows (n = 211), EEG technologists (n = 128), attending neurologists (n = 111), adult neurology residents (n = 227), child neurology residents (n = 108), medical students (n = 24), attending non-neurologists (n = 18), and others (n = 54). Mean overall scores (95% CI) were 82% (77–86) (clinical neurophysiologists), 81% (79–83) (clinical fellows), and 72% (70–73) (adult and child neurology residents). Experts were more confident than clinical fellows in all categories but sleep structures. Experts and clinical fellows were more confident than residents in all 4 categories. Among residents, accuracy and LOC increased as a function of prior EEG weeks of training. Accuracy improved from 67% (baseline/no prior EEG training) to 77% (>12 prior EEG weeks). More than 8 weeks of EEG training was needed to reach accuracy comparable with clinical neurophysiologists on this rEEG examination. Increase in LOC was slower and less robust than increase in accuracy. All but 3 questions had a high discrimination index (>0.25).

**Discussion:**

This online, competency-based rEEG examination, mapped to a published EEG curriculum, has excellent psychometrics and differentiates experienced EEG readers from adult and child neurology residents. This online tool has the potential to improve resident EEG education worldwide.

## Introduction

EEG is one of the cornerstones in the diagnosis and management of patients with seizures and epilepsy. From a diagnostic standpoint, EEG has a critical role in establishing a diagnosis of epilepsy—for example, in cases where paroxysmal events are of moderate clinical suspicion for epileptic seizures^1,2^ or in cases where there is a single unprovoked epileptic seizure.^3^ In addition, EEG is helpful in classifying epilepsy and guiding workup and treatment.^1^ Finally, EEG can assist in understanding prognosis and stratifying risk of seizure recurrence upon antiseizure drug withdrawal.^1,4^

The important clinical role of EEG requires accurate and reliable EEG interpretation. If this condition is not met, the care of patients with seizures and epilepsy is compromised and deleterious outcomes may ensue.^5^ It is, therefore, critical to ensure that all those who read EEG in clinical practice are fully competent. In many countries, such as the United States and select European countries, the workforce of EEG readers includes neurologists without postresidency training in clinical neurophysiology/EEG or epilepsy.^6-9^ This practice dictates that adult and child neurology residency training provides optimal EEG training, thus allowing residents to achieve EEG competency by the time of graduation.

Prior studies suggest that a significant portion of neurology residents graduate from residency without achieving EEG competency.^10-14^ There have been multiple efforts to understand this educational gap and address barriers to resident EEG education. One of these efforts sought to standardize the resident EEG learning process by the curation of curricular objectives for teaching and assessing routine EEG (rEEG) interpretation.^15^ These curricular objectives consist of a list of “must-know” rEEG findings for adult and child neurology residents.^15^ Notably, interpretation of other types of EEGs, such as those performed in critically ill patients, was not addressed. In this study, we used this curriculum framework^15^ to develop and validate an online, multiple-choice rEEG examination for adult and child neurology trainees—which may be used for both formative and summative assessment.

## Methods

**Figure 1:**
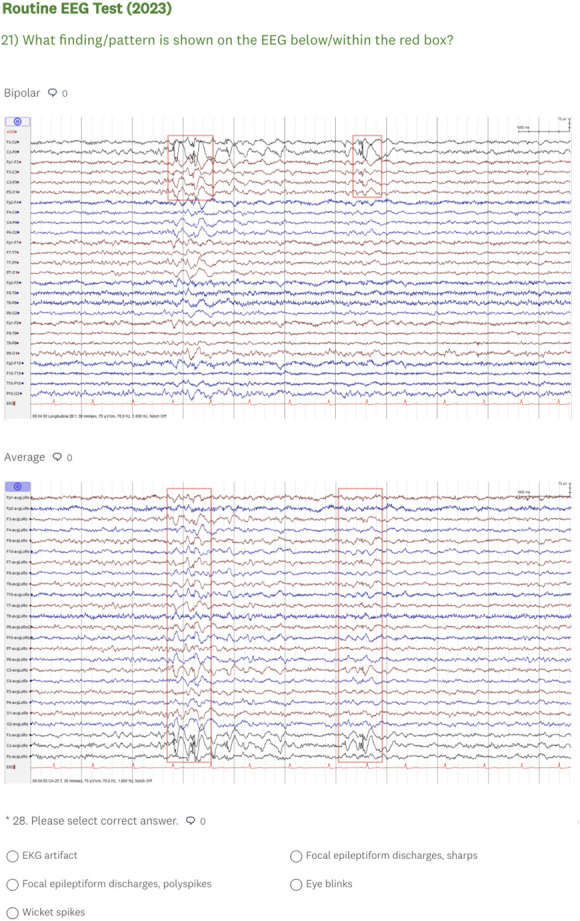
Representative Screenshot of the Online Routine EEG Examination

We developed an online rEEG examination (Figure 1) based on a previously published rEEG curriculum content map.^15^ The examination was hosted on Survey Monkey and consisted of a brief survey and 30 multiple-choice questions covering 4 EEG domains: normal (n = 8/30), abnormal (n = 7/30), normal variants (n = 7/30), and artifacts (n = 8/30) (Table 1). Multiple-choice questions tested rEEG findings considered high yield by a large group of multinational EEG/epilepsy experts based on the importance for adult and child neurology residents to learn these findings during residency training.^15^

**Table 1:**
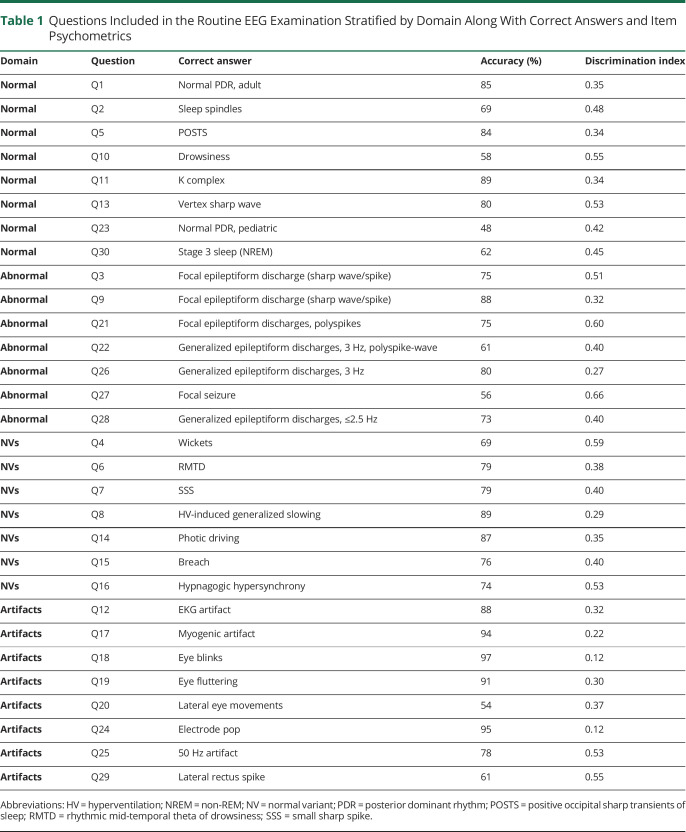
Questions Included in the Routine EEG Examination Stratified by Domain Along With Correct Answers and Item Psychometrics

Domain	Question	Correct answer	Accuracy (%)	Discrimination index
Normal	Q1	Normal PDR, adult	85	0.35
Normal	Q2	Sleep spindles	69	0.48
Normal	Q5	POSTS	84	0.34
Normal	Q10	Drowsiness	58	0.55
Normal	Q11	K complex	89	0.34
Normal	Q13	Vertex sharp wave	80	0.53
Normal	Q23	Normal PDR, pediatric	48	0.42
Normal	Q30	Stage 3 sleep (NREM)	62	0.45
Abnormal	Q3	Focal epileptiform discharge (sharp wave/spike)	75	0.51
Abnormal	Q9	Focal epileptiform discharge (sharp wave/spike)	88	0.32
Abnormal	Q21	Focal epileptiform discharges, polyspikes	75	0.60
Abnormal	Q22	Generalized epileptiform discharges, 3 Hz, polyspike-wave	61	0.40
Abnormal	Q26	Generalized epileptiform discharges, 3 Hz	80	0.27
Abnormal	Q27	Focal seizure	56	0.66
Abnormal	Q28	Generalized epileptiform discharges, ≤2.5 Hz	73	0.40
NVs	Q4	Wickets	69	0.59
NVs	Q6	RMTD	79	0.38
NVs	Q7	SSS	79	0.40
NVs	Q8	HV-induced generalized slowing	89	0.29
NVs	Q14	Photic driving	87	0.35
NVs	Q15	Breach	76	0.40
NVs	Q16	Hypnagogic hypersynchrony	74	0.53
Artifacts	Q12	EKG artifact	88	0.32
Artifacts	Q17	Myogenic artifact	94	0.22
Artifacts	Q18	Eye blinks	97	0.12
Artifacts	Q19	Eye fluttering	91	0.30
Artifacts	Q20	Lateral eye movements	54	0.37
Artifacts	Q24	Electrode pop	95	0.12
Artifacts	Q25	50 Hz artifact	78	0.53
Artifacts	Q29	Lateral rectus spike	61	0.55

Abbreviations: HV = hyperventilation; NREM = non-REM; NV = normal variant; PDR = posterior dominant rhythm; POSTS = positive occipital sharp transients of sleep; RMTD = rhythmic mid-temporal theta of drowsiness; SSS = small sharp spike.

In the normal domain, we tested the top 10 high-yield EEG findings.^15^ We used 1 question (question 10) to test 3 EEG findings associated with drowsiness: slowing of the posterior dominant rhythm, diffuse irregular delta-theta slowing of the background, and slow roving lateral eye movements.

In the artifacts domain, we tested the top 7 high-yield EEG findings in addition to the ninth EEG finding on the rank list.^15^ We skipped the eighth EEG finding on the list (pulse artifact) because this finding is less often seen in rEEG clinical practice. We arbitrarily elected to test 50 Hz artifact rather than 60 Hz artifact given their electrographic similarity; however, we note that they are typically seen in Europe and North America, respectively.

In the normal variants domain, we tested the top 5 high-yield EEG findings in addition to the seventh and eighth EEG findings on the rank list.^15^ We skipped the sixth EEG finding (hypnopompic hypersynchrony) because this finding is also less often seen in rEEG clinical practice and because of its electrographic similarity with hypnagogic hypersynchrony, which was included in the test.

In the abnormal domain, we tested the top 5 high-yield EEG findings in addition to the seventh and eighth EEG findings on the rank list.^15^ We skipped the sixth EEG finding (generalized seizure, absence) because this EEG pattern was electroencephalographically represented by the first EEG finding on the list (generalized epileptiform discharge, 3 Hz).

The survey included questions regarding participants' country of origin, profession (medical student, adult neurology resident, child neurology resident, EEG/epilepsy clinical fellow, or other), year of training (if applicable), number of weeks dedicated to EEG learning during training (0, 1–4, 5–8, 9–12, or >12) (if applicable), and level of confidence (LOC, 5-point Likert scale: from “not at all confident” to “very confident”) identifying 4 categories of EEG findings independently/without supervision: states of wakefulness/sleep, sleep structures, normal variants, and artifacts. The survey did not include a question addressing participants' country of practice/training.

The “other” group, under profession, required a free text response to characterize respondents' profession. We used these free text responses to divide the “other” category into clinical neurophysiologists (aka EEG experts), EEG technologists, attending neurologists, attending non-neurologists, and others. The latter category comprised those “other” respondents whose free text answers were either nonspecific (e.g., “MD,” “clinician,” “attending,” “physician,” “consultant,” “medical graduate,” “academic,” “staff physician,” “junior consultant,” “neurology,” or “research fellow”) or corresponded to other professions (e.g., epilepsy research fellow, neurophysiology residents and scientists, pediatric neurology/neurogenetics fellow/resident, neurosurgery and neurology clinical fellow, psychiatry resident, scientists, sleep medicine PhD student, or medical assistant/nursing staff).

Each question included a deidentified, 10- to 20-second EEG image displayed in 2 montages (bipolar and common average). These EEGs were obtained from 2 authors' (F.A.N., S.B.) personal collections and were deemed unequivocal examples of the findings tested by 5 EEG experts authoring this work (F.A.N., R.M., S.R., W.O.T., S.B.).

All 30 questions had 5 answer choices. All but 2 questions had 1 correct and 4 incorrect answers. Questions 3 and 9 had 2 correct answers: “focal epileptiform discharge, spike” and “focal epileptiform discharge, sharp.” We considered both answer choices as correct because it was virtually impossible for respondents to estimate the sharp transient duration without an interactive ruler, hence being unable to correctly differentiate a sharp wave (duration of 70–200 milliseconds) from a spike (duration of <70 milliseconds). Incorrect answers were judiciously selected to represent reasonable and important electroencephalographic differentials of the EEG finding tested.

Questions were reviewed and improved, and correct and incorrect answers were adjudicated by 5 EEG experts (F.A.N., R.M., S.R., W.O.T., S.B.). The rEEG examination was disseminated on social media from the authors' personal accounts (F.A.N., R.M., R.S., S.B.) and through email and social media by the International League Against Epilepsy (ILAE). Data were collected from June 2022 to December 2022.

After data collection and study completion, the authors created a new version of the rEEG examination with instant feedback for self-paced online formative assessment. Feedback is given for all answer choices—correct and incorrect ones. Examination content remained unchanged, except for questions 3 and 9 where we replaced EEG images with easier-to-visualize examples of a sharp wave and a spike, respectively. In addition, we included a more detailed scale, which was placed closer to the sharp wave/spike. These modifications, derived from what we learned from our study results, were made to ensure that respondents can measure the sharp transient duration and, therefore, determine whether the focal epileptiform discharge is a sharp wave (70–200 milliseconds) or a spike (<70 milliseconds). The formative assessment version of the rEEG examination with feedback will be made publicly available at the time of publication of this study and integrated into the online educational portfolio of the ILAE Academy (eAppendix 1, links.lww.com/NE9/A42).

### Statistical Analysis

Item analysis (difficulty/accuracy and discrimination index) was performed using the Statistical Analysis System. Regression models were used to compare 3 groups (clinical neurophysiologists, EEG/epilepsy clinical fellows, and adult and child neurology residents) regarding accuracy: overall and stratified by each test domain (normal, abnormal, normal variants, and artifacts). Logistic regression models were used to compare these 3 groups regarding LOC identifying 4 categories of EEG findings (states of wakefulness/sleep, sleep structures, normal variants, and artifacts) independently/without supervision. Finally, we examined the relationships between the number of weeks of prior EEG training among (adult and child) neurology residents and their (1) mean overall scores and (2) LOC identifying the 4 categories of EEG findings. In the former analysis, we compared the mean overall scores in each group based on prior EEG weeks of training (0, 1–4, 5–8, 9–12, and >12) with the mean overall score among clinical neurophysiologists. Statistical significance was assumed for *p* < 0.05.

### Standard Protocol Approvals, Registrations, and Patient Consents

Completion of the online EEG test was voluntary, and informed consent was verified when all respondents to the survey provided permission for participation in the first question of the rEEG examination (eAppendix 2, links.lww.com/NE9/A43). No identifying information was collected from participants. This study was approved by the institutional review board and data safety officer at the senior author's (S.B.) institution (EMN-2023-02938).

### Data Availability

Study data will be made available by request from any qualified investigator. The rEEG examination with (formative) and without (summative) feedback will be publicly available on the internet.

## Results

### Overall Test Accuracy

**Figure 2:**
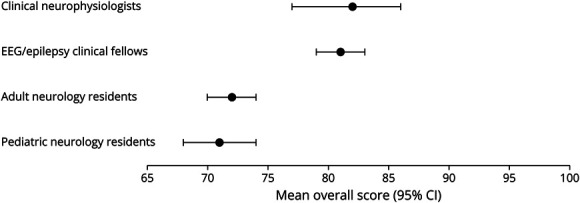
Mean Overall Scores Among Clinical Neurophysiologists, EEG/Epilepsy Clinical Fellows, Adult Neurology Residents, and Child Neurology Residents

There was a total of 2,080 responses. We included those that were complete and whose respondents provided consent (n = 922/2,080). Respondents and their respective mean percentage overall scores (mean [95% CI]) were as follows: clinical neurophysiologists (n = 41; 82 [77–86]), EEG/epilepsy clinical fellows (n = 211; 81 [79–83]), adult neurology residents (n = 227; 72 [70–74]), and child neurology residents (n = 108; 71 [68–74]). These data are presented in Figure 2.

Additional study groups and their respective mean percentage overall scores (mean [95% CI]) were as follows: EEG technologists (n = 128; 81 [79–84]), attending neurologists (n = 111; 78 [76–81]), medical students (n = 24; 70 [60–80]), attending non-neurologists (n = 18; 56 [44–67]), and others (n = 54; 77 [72–82]). These data are presented in eFigure 1 (links.lww.com/NE9/A45).

### Test Content Validity

Test content maps to a published EEG curriculum^15^ and as such is divided into 4 domains: normal, abnormal, normal variants, and artifacts (Table 1). Within each domain, we tested EEG findings with the highest educational yield.^15^

### Test Psychometric Validity

The accuracy and discrimination index for each question are summarized in Table 1. All but 3 questions (questions 17, 18, and 24) had a discrimination index of at least 0.25.

### Test Consequential Validity

We performed further analyses in the group of adult and child neurology residents for their number of prior weeks dedicated to EEG learning during training and (1) mean overall scores and (2) LOC (5-point scale) identifying 4 categories of EEG findings independently/without supervision: states of wakefulness/sleep, sleep structures, normal variants, and artifacts. Notably, 1 adult neurology resident included in the study did not provide his/her number of weeks of prior EEG training. Therefore, we included a total of 344 adult and child neurology residents in this set of analyses.

### Mean Overall Scores vs Number of Weeks of Prior EEG Training

The mean overall percentage scores (mean [95% CI]) for adult and child neurology residents (n = 334) stratified by the number of prior EEG weeks of training were 67 (59–74) at baseline/no prior EEG training, 66 (62–69) for 1–4 weeks of prior EEG training, 72 (69–75) for 5–8 weeks, 73 (69–77) for 9–12 weeks, and 77 (74–79) for >12 weeks. The mean overall percentage score in each group was compared with the mean overall percentage score (mean [95% CI]) among clinical neurophysiologists (n = 41, 82 (77–86)).

**Figure 3:**
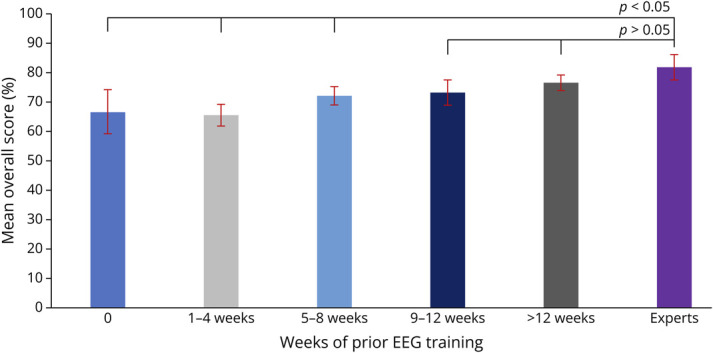
Mean Overall Scores Stratified by Number of Weeks of Prior EEG Training Among Adult and Child Neurology Residents (n = 334)

There was a statically significant difference between mean overall scores among clinical neurophysiologists and neurology residents with (1) 0 (*p* = 0.0045), (2) 1–4 weeks (*p* < 0.0001), and (3) 5–8 weeks (*p* = 0.0088) of prior EEG training. Conversely, there was no statistically significant difference between mean overall scores among clinical neurophysiologists and neurology residents with 9–12 weeks (*p* = 0.0641) or >12 weeks (*p* = 0.3495) of prior EEG training. These analyses are demonstrated in Figure 3.

### LOC in EEG Reading Without Supervision vs Number of Weeks of Prior EEG Training

We calculated the percentage of adult and child neurology residents in each subgroup (0, 1–4, 5–8, 9–12, and >12 weeks of prior EEG training) who reported being “very confident” (highest LOC in our 5-point scale) identifying 4 categories of EEG findings independently/without supervision: states of wakefulness/sleep, sleep structures, normal variants, and artifacts.

**Figure 4:**
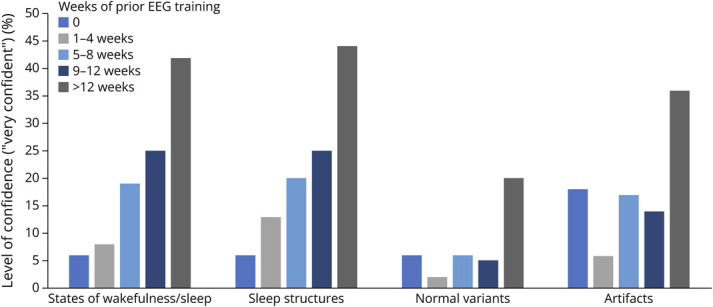
Level of Confidence Stratified by Number of Weeks of Prior EEG Training Among Adult and Child Neurology Residents (n = 334) “Very Confident”

In the states of wakefulness/sleep category, this percentage was 6 (no prior EEG training), 8 (1–4 weeks), 19 (5–8 weeks), 25 (9–12 weeks), and 42 (>12 weeks). In the sleep structures category, this percentage was 6 (no prior EEG training), 13 (1–4 weeks), 20 (5–8 weeks), 25 (9–12 weeks), and 44 (>12 weeks). In the normal variants category, this percentage was 6 (no prior EEG training), 2 (1–4 weeks), 6 (5–8 weeks), 5 (9–12 weeks), and 20 (>12 weeks). In the artifacts category, this percentage was 18 (no prior EEG training), 6 (1–4 weeks), 17 (5–8 weeks), 14 (9–12 weeks), and 36 (>12 weeks). These data are presented in Figure 4.

### Additional Group Analyses, Scores, and LOCs

We further analyzed accuracy of the 3 major groups: clinical neurophysiologists (n = 41), EEG/epilepsy clinical fellows (n = 211), and adult and child neurology residents (n = 335). Mean overall percentage scores and mean percentage scores in each EEG domain for each major group are summarized in Table 2. Comparisons of mean overall and domain-specific scores among these 3 major groups are discussed below and summarized in eAppendix 3 (links.lww.com/NE9/A44), respectively.

**Table 2:**
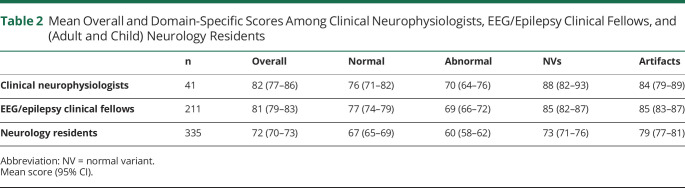
Mean Overall and Domain-Specific Scores Among Clinical Neurophysiologists, EEG/Epilepsy Clinical Fellows, and (Adult and Child) Neurology Residents

	n	Overall	Normal	Abnormal	NVs	Artifacts
Clinical neurophysiologists	41	82 (77–86)	76 (71–82)	70 (64–76)	88 (82–93)	84 (79–89)
EEG/epilepsy clinical fellows	211	81 (79–83)	77 (74–79)	69 (66–72)	85 (82–87)	85 (83–87)
Neurology residents	335	72 (70–73)	67 (65–69)	60 (58–62)	73 (71–76)	79 (77–81)

Abbreviation: NV = normal variant.

Mean score (95% CI).

Regarding mean overall scores, there was a statistically significant difference between clinical neurophysiologists and neurology residents (*p* = 0.0002) and between EEG/epilepsy clinical fellows and neurology residents (*p* < 0.0001). There was no statistically significant difference between clinical neurophysiologists and EEG/epilepsy clinical fellows (*p* = 0.9347).

We also compared LOC identifying 4 categories of EEG findings independently/without supervision among the 3 major groups. In the states of wakefulness/sleep, normal variants, and artifacts categories, clinical neurophysiologists were more confident than EEG/epilepsy clinical fellows who were more confident than neurology residents. In the sleep structures category, clinical neurophysiologists were as confident as EEG/epilepsy clinical fellows, but both groups were more confident than neurology residents. These data are summarized in Table 3.

**Table 3:**
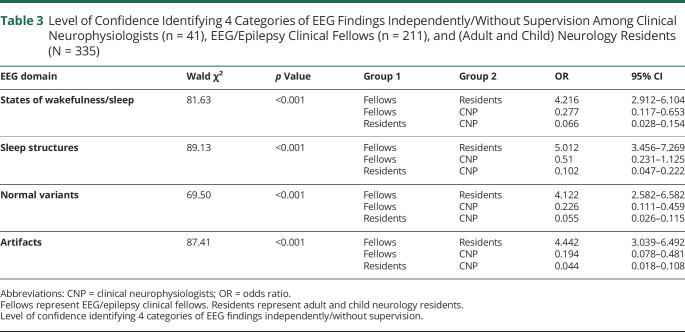
Level of Confidence Identifying 4 Categories of EEG Findings Independently/Without Supervision Among Clinical Neurophysiologists (n = 41), EEG/Epilepsy Clinical Fellows (n = 211), and (Adult and Child) Neurology Residents (N = 335)

EEG domain	Wald χ^2^	*p* Value	Group 1	Group 2	OR	95% CI
States of wakefulness/sleep	81.63	<0.001	Fellows Fellows Residents	Residents CNP CNP	4.216 0.277 0.066	2.912–6.104 0.117–0.653 0.028–0.154
Sleep structures	89.13	<0.001	Fellows Fellows Residents	Residents CNP CNP	5.012 0.51 0.102	3.456–7.269 0.231–1.125 0.047–0.222
Normal variants	69.50	<0.001	Fellows Fellows Residents	Residents CNP CNP	4.122 0.226 0.055	2.582–6.582 0.111–0.459 0.026–0.115
Artifacts	87.41	<0.001	Fellows Fellows Residents	Residents CNP CNP	4.442 0.194 0.044	3.039–6.492 0.078–0.481 0.018–0.108

Abbreviations: CNP = clinical neurophysiologists; OR = odds ratio.

Fellows represent EEG/epilepsy clinical fellows. Residents represent adult and child neurology residents.

Level of confidence identifying 4 categories of EEG findings independently/without supervision.

### Questions With Highest and Lowest Accuracy

The top 10 questions with overall lowest accuracy are listed in eTable 1 (links.lww.com/NE9/A46). The top 10 questions with overall highest accuracy are listed in eTable 2.

## Discussion

We developed a competency-based, online rEEG examination based on a published EEG curriculum for adult and child neurology residents^15^ using expert-certified unequivocal EEG examples of findings considered educationally high yield (aka “must-know”) for adult and child neurology residents. The examination was completed by approximately 1,000 participants across the globe including clinal neurophysiologists (n = 41), EEG/epilepsy clinical fellows (n = 211), and adult and child neurology residents (n = 335). Our results support that this examination has excellent overall psychometrics and accurately differentiates experienced EEG readers (attending neurologists, EEG technologists, EEG/epilepsy clinical fellows, and clinical neurophysiologists) from adult and child neurology residents and attending non-neurologists based on accuracy.

The examination's ability to distinguish neurology residents from experienced EEG readers is promising because it allows trainees to undergo a tangible, competency-based model of assessment. The utilization of this examination becomes even more appealing because a large portion of neurology residency programs in the United States (64%)^14^ and in Europe (47%)^8^ do not currently use objective measures to assess EEG competency. Importantly, this learner-centered, competency-based model has been guiding medical education over the past decades^16^ and is arguably an optimal framework in the field of EEG to ensure that neurology residents achieve competency by graduation.^15^

Specifically concerning the group of adult and child neurology residents in this study, we learned that their overall accuracy in identifying “must-know” EEG findings for neurology residents^15^ does not become statistically comparable with the mean overall accuracy among clinical neurophysiologists until after residents have had more than 8 weeks of EEG training. Given that the examination includes unequivocal examples of EEG findings considered “must-know” for adult and child neurology residents,^15^ we believe that the mean overall accuracy among clinical neurophysiologists (mean 82%, 95% CI 77–86) should be the target score for neurology trainees by the time they complete residency training.

We, therefore, recommend that neurology residents undergo compulsory extended EEG education of more than 8 weeks of EEG training before graduation. Based on our results, this is required to ensure that neurology graduates attain minimal competency to allow them to accurately identify unequivocal examples of “must-know” rEEG findings. Establishing an ideal minimum number of weeks of EEG training is key in training environments where there are considerable time-based requirements, such as residency training. We would like to highlight that our rEEG examination contained only 1 example—deemed unequivocal and clearly representative—of each EEG finding tested, thereby not including the full array of nuances and subtleties that may be featured in each EEG finding. It is, therefore, reasonable to conclude that trainees require a greater number of EEG weeks to be able to be fully competent in reading rEEGs in clinical practice. This additional training may be undertaken either during residency or, more realistically, during fellowship.

Ensuring that these recommendations are implemented is crucial, especially in countries where neurologists without postresidency training in clinical neurophysiology/EEG or epilepsy typically read EEGs in clinical practice—such as the United States^6,7,9^ and select European countries.^8^ In the United States, for instance, it has been shown that EEG is the most common procedure performed by neurologists (∼60%).^6^ Similarly, survey data on the US child neurology workforce have shown that more than half of child neurology/neurodevelopmental disabilities specialists who manage patients with epilepsy and read EEGs had no formal EEG training other than what was received during residency.^9^ Nevertheless, EEG training is not a requirement for graduation in neurology residencies in the United States,^17,18^ leading to some programs requiring no EEG training altogether.^14^

This target of more than 8 weeks of EEG training is higher than the mean number of EEG weeks required to graduate in neurology programs in the United States (6.8 weeks, range 0–16)^14^ and in European countries where general neurologists are not among the providers who typically read EEGs in clinical practice (7.4 weeks).^8^ Nonetheless, it is comparable with the mean number of EEG weeks required to graduate in European countries where general neurologists are either among the providers or the only providers who read EEGs in clinical practice (9.2 weeks).^8^

In this study, we also found that it required more than 12 weeks of prior EEG training for at least 36% of residents to report feeling very confident identifying EEG findings related to states of wakefulness/sleep, sleep structures, and artifacts without supervision. Conversely, a minority of residents (20%) felt very confident identifying EEG findings in the category of normal variants despite having had more than 12 weeks of prior EEG training.

The ramifications of these data are twofold. First, we identified a mismatch between residents' increase in accuracy and LOC as they undergo EEG training in residency. The increase in LOC seems to be slower than the increase in accuracy. Second, although they reach acceptable accuracy with more than 8 weeks of EEG training, their LOC identifying EEG findings without supervision increases slowly and does not overpass 50% even with more than 12 weeks of training. Notably, this slow and suboptimal increase in LOC is particularly evident in the normal variants category with an end LOC of 20% for those residents with more than 12 weeks of prior EEG training.

Finally, we learned that clinical neurophysiologists and EEG/epilepsy clinical fellows performed similarly but greater than adult and neurology residents in the examination addressing “must-know” EEG findings for adult and child neurology residents^15^ both overall as well as in its normal, abnormal, and normal variants domains. In the artifacts domain, however, there was no significant difference in accuracy among the 3 groups, except for EEG/epilepsy clinical fellows performing greater than adult and child neurology residents. In addition, we learned that the accuracy among attending non-neurologists was significantly lower than experienced EEG readers and adult and child neurology residents.

Regarding LOC identifying 4 categories of EEG findings without supervision, clinical neurophysiologists were more confident than EEG/epilepsy clinical fellows who were more confident than adult and child neurology residents in the categories of states of wakefulness/sleep, normal variants, and artifacts. In the sleep structures category, clinical neurophysiologists were as confident as EEG/epilepsy clinical fellows, and both groups were more confident than adult and child neurology residents.

These data highlight that although clinical neurophysiologists performed as well as EEG/epilepsy clinical fellows on this rEEG examination, the former group had a relatively higher LOC compared with the latter group. This discrepancy may be explained by the fact that the rEEG examination was targeted at a trainee level (low overall difficulty level for experienced EEG readers). Consequently, both clinical neurophysiologists and EEG/epilepsy clinical fellows performed similarly. However, the overall LOC obtained for both groups was unrelated to the rEEG examination itself and rather referred to their overall perceptions of identifying EEG findings independently.

Regarding psychometric validity, all but 3 questions in the examination had a discrimination index of >0.25. These data support the competency-based assessment purpose of the examination wherein experienced EEG readers are separated from nonexperienced EEG readers based on accuracy (aka EEG proficiency and skills). The 3 questions with a lower discrimination index, and higher overall accuracy (>90%), are also vital because they addressed important EEG findings (electrode pop, eye blinks, and myogenic artifact) that must be mastered at an early stage of training by all EEG readers, irrespective of their experience.

Our study has several limitations. First, owing to the anonymous nature of the examination, there may have been more than 1 recorded answer for the same participant. Similarly, we could not confirm participants' profession or level of training or past EEG experience (if neurology residents). We did not obtain participants' country of practice/training although, given that our rEEG examination was disseminated on the internet by the authors and ILAE, we assume that our participants were from many different countries. This limitation should be taken into account upon interpreting our results and recommendations because adult and child neurology residency as well as EEG/epilepsy fellowship training vary considerably across countries.^8,19,20^ Second, for participants who were neurology residents, we were provided with a number of weeks of prior EEG training only. We acknowledge that EEG training relies greatly on the quality of education in addition to the quantity. We believe this limitation may have been mitigated by the large number of neurology residents included in the study. Third, the examination was “open book,” and participants did not have time constraints to complete the test. These characteristics may have influenced their scores. Fourth, although the EEG examples included in the test were believed to be unequivocal per 5 authors, who are EEG experts, we identified expert inter-rater variability (IRV) based on the scores of other clinical neurophysiologists/EEG experts who also completed the examination. This phenomenon is a known limitation of expert EEG interpretation using qualitative visual analysis.^21^ Moreover, the examination included only 1 example of each EEG finding tested, although, in clinical EEG practice, each type of EEG finding may be represented by a multitude of nonidentical waveforms. Fifth, our study was not designed to assess retention of skill because all respondents were tested only once.

Future directions for our research include improving our competency-based rEEG examination by adding more examples of each of the “must-know” findings, conceivably of different levels of difficulty (based on the level of expert agreement). Furthermore, we plan to have our EEG examples labeled by a larger cohort of experts, thereby expanding the expert consensus-based gold standard. Moreover, rather than showing EEG examples as static images, we plan to create an online, publicly accessible EEG platform where learners can interact with EEGs shown, for example, by changing the montage and modifying sensitivity. We will also include longer EEGs including findings of interest where learners are able to scroll and find those EEG patterns before interpreting them. Finally, it would be helpful to have similar competency-based EEG examinations focused on other types of EEGs, such as critical care EEG, epilepsy monitoring unit EEG, and intracranial EEG.

Another area that requires further research is the unexpectedly large expert inter-rater variability in the rEEG examination. Increased expert IRV is a well-known phenomenon in the realm of interictal epileptiform discharge (IED) identification^21,22^ and seizures and rhythmic and periodic pattern identification.^23^ Nonetheless, expert IRV has not been fully explored in other rEEG findings such as abnormal findings other than IEDs, normal findings, normal variants, and artifacts.

We believe our rEEG examination may be used by educators, administrators, and regulators in several ways. In its version with feedback, it can be used as a self-paced online teaching tool (formative assessment) where the highest yield “must-know” rEEG findings for adult and child neurology residents are included. In its version without feedback, it can be used as a competency-based assessment tool. This tool can be used longitudinally throughout residency training, where a target accuracy would depend on the number of EEG weeks residents have had. Educators may tailor individual and collective EEG educational activities based on residents' examination scores. This tool can also be used at the time of residency training completion, where an accuracy comparable with expert-level accuracy would be a surrogate of trainee EEG competency within the realm of rEEG. Finally, on implementation of our rEEG examination on the ILAE Academy online learning platform, learners will be able to use this tool as a formative and summative assessment concomitantly.

Finally, we recommend that adult and neurology residents undergo more than 8 weeks (ideally >12 weeks) of EEG training to ensure minimal rEEG competency by the time they graduate. We also suggest educators consider accounting for the observation that residents typically improve their accuracy before their LOC increases throughout their training—especially regarding EEG normal variants. We believe that the rEEG examination will be a useful resource in EEG education and that it will help EEG education teaching and assessment become more objective and standardized.

## Appendix Authors

**Table:**

Name	Location	Contribution
Fábio A. Nascimento, MD	Division of Epilepsy, Department of Neurology, Washington University School of Medicine, St. Louis, MO; Department of Neurology, Massachusetts General Hospital, Harvard Medical School, Boston	Drafting/revision of the manuscript for content, including medical writing for content; study concept or design; analysis or interpretation of data
Hong Gao, MD	Department of Internal Medicine, Wake Forest University School of Medicine, Winston-Salem, NC	Drafting/revision of the manuscript for content, including medical writing for content; analysis or interpretation of data
Roohi Katyal, MD	Division of Epilepsy, Department of Neurology, Louisiana State University Health Shreveport	Drafting/revision of the manuscript for content, including medical writing for content; analysis or interpretation of data
Rebecca Matthews, MD	Department of Neurology, Emory University School of Medicine, Atlanta, GA	Drafting/revision of the manuscript for content, including medical writing for content; analysis or interpretation of data
Samantha V. Yap, BS	Department of Neurology, Massachusetts General Hospital, Harvard Medical School, Boston, MA	Drafting/revision of the manuscript for content, including medical writing for content; analysis or interpretation of data
Stefan Rampp, MD	Department of Neurosurgery, University Hospital Erlangen, Erlangen; Department of Neurosurgery, University Hospital Halle (Saale), Germany	Drafting/revision of the manuscript for content, including medical writing for content; analysis or interpretation of data
William O. Tatum, DO	Department of Neurology, Mayo Clinic, Jacksonville, FL	Drafting/revision of the manuscript for content, including medical writing for content; analysis or interpretation of data
Roy E. Strowd III, MD, MEd, MS	Department of Neurology, Wake Forest University School of Medicine, Winston-Salem, NC	Drafting/revision of the manuscript for content, including medical writing for content; study concept or design; analysis or interpretation of data
Sándor Beniczky, MD, PhD	Department of Clinical Neurophysiology, Danish Epilepsy Center, Dianalund and Aarhus University Hospital; Department of Clinical Medicine, Aarhus University, Aarhus, Denmark	Drafting/revision of the manuscript for content, including medical writing for content; study concept or design; analysis or interpretation of data

## Glossary

IED: interictal epileptiform discharge
ILAE: International League Against Epilepsy
IRV: inter-rater variability
LOC: level of confidence
rEEG: routine EEG
